# Trends in the Prevalence of Exposure to e-Cigarette Aerosol in Public Places Among US Middle and High School Students, 2015 to 2018

**DOI:** 10.1001/jamanetworkopen.2019.10184

**Published:** 2019-08-28

**Authors:** Andy S. L. Tan, Cabral A. Bigman, Susan Mello, Ashley Sanders-Jackson

**Affiliations:** 1 Center for Community Based Research, Division of Population Sciences, Dana-Farber Cancer Institute, Boston, Massachusetts; 2 Department of Social and Behavioral Sciences, Harvard T.H. Chan School of Public Health, Boston, Massachusetts; 3 Department of Communication, College of Liberal Arts and Sciences, University of Illinois Urbana-Champaign, Urbana; 4 College of Arts, Media and Design, Department of Communication Studies, Northeastern University, Boston, Massachusetts; 5 College of Communication Arts and Science, Department of Advertising and Public Relations, Michigan State University, East Lansing

## Abstract

This survey study examines trends in and factors associated with exposure to secondhand smoke from combusted tobacco and secondhand aerosol from electronic cigarettes (e-cigarettes) among US youth from 2015 to 2018.

## Introduction

Approximately one-quarter of US youth were exposed to secondhand electronic cigarette (e-cigarette) aerosols between 2015 and 2017.^1^ Given the rapid increase in vaping and the popularity of pod-based e-cigarettes among youth,^2^ this survey study examines trends in and factors associated with exposure to secondhand smoke (SHS) from combusted tobacco and secondhand aerosol (SHA) from e-cigarettes among US youth.

## Methods

Data were from the National Youth Tobacco Survey from 2015 to 2018. Sample sizes (overall response rates) were 17 711 (63.4%) in 2015, 20 675 (71.6%) in 2016, 17 872 (68.1%) in 2017, and 20 189 (68.2%) in 2018. Response rates were computed using the product of school-level participation and student-level participation. The US Centers for Disease Control and Prevention institutional review board approved the data collection protocol. Parents used either active or passive permission forms. Participation among students was voluntary, and anonymous and oral consent was obtained from students to ensure anonymity.^2^ This analysis is a secondary analysis of deidentified, publicly available data; therefore, no ethics approval was sought.

Participants were asked how often they breathed smoke from someone who was smoking tobacco products and breathed vapor from someone using an e-cigarette in indoor (eg, school building, store, restaurant, and sports arena) or outdoor (eg, school grounds, parking lot, stadium, and park) public places in the past 30 days. Response options were 0, 1 or 2, 3 to 5, 6 to 9, 10 to 19, 20 to 29, or all 30 days. We recoded responses as no exposure (0 days) vs exposure (≥1 day). Potential factors included sex, school type, race/ethnicity (as classified by the National Youth Tobacco Survey), speaking non-English language at home, e-cigarette use, past-30-day use of other tobacco products, living with someone who used e-cigarettes, and living with someone who used other tobacco products.

We compared the prevalence of exposure to SHS and SHA annually using bivariate logistic regression of SHS and SHA exposure with 2018 data by fitting multiple logistic regressions. All analyses were conducted using Stata version 14 (StataCorp) and weighted to account for the complex survey design and to be representative of the US middle and high school population. Statistical significance was set at *P* < .05, and all tests were 2-tailed.

## Results

Between 2015 and 2018, approximately half of US middle school and high school students reported SHS exposure in the preceding 30 days, with a significant downward trend in 2017 and 2018 (2015, 52.6%; 2016, 53.4%; 2017, 50.5%; 2018, 48.7%) (Figure). Prevalence of SHA exposure increased from approximately 1 of 4 students between 2015 and 2017 to 1 of 3 students in 2018, with a significant upward trend in 2018 (2015, 25.2%; 2016, 26.5%; 2017, 25.6%; 2018, 33.2%) (Figure).

**Figure.**
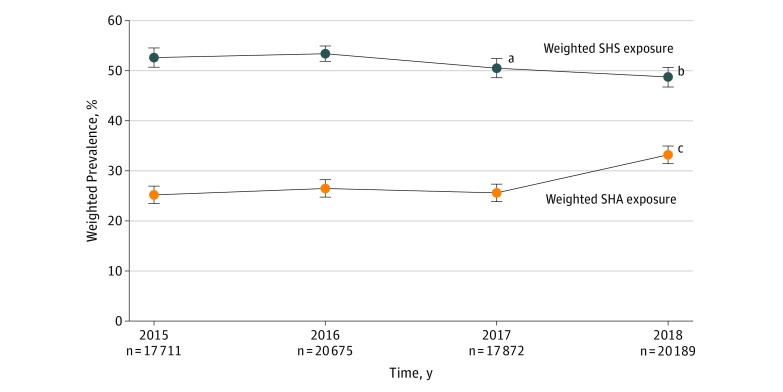
Trends in Prevalence of Secondhand Smoke (SHS) and Secondhand Aerosol (SHA) Exposure in Public Places Among US Middle and High School Students Whiskers represent 95% CIs for each prevalence estimate. Prevalence estimates and 95% CIs were based on weighted analyses to be representative of the US middle and high school student population. The number of missing cases for SHS and SHA exposure items were, respectively, 809 (4.6%) and 791 (4.5%) in 2015, 1031 (5.0%) and 1063 (5.1%) in 2016, 819 (4.6%) and 811 (4.5%) in 2017, and 1015 (5.0%) and 989 (4.9%) in 2018. ^a^Significant difference between SHS exposure in 2017 and exposure in 2016. ^b^Significant difference between SHS exposure in 2018 and exposure in 2015 and 2016. ^c^Significant difference between SHA exposure in 2018 and exposure in 2015, 2016, and 2017.

Based on 2018 data, young women (SHS: adjusted odds ratio [aOR], 1.96; 95% CI, 1.80-2.12; SHA: aOR, 1.68; 95% CI, 1.52-1.86), non-Hispanic white individuals (non-Hispanic black, SHS: aOR, 0.64; 95% CI, 0.53-0.77; SHA: 0.47; 95% CI, 0.39-0.56), participants who used e-cigarettes ever (SHS: aOR, 1.70; 95% CI, 1.52-1.92; SHA: aOR, 2.30; 95% CI, 2.02-2.62) and in the past 30 days (SHS: aOR, 1.88; 95% CI, 1.64-2.17; SHA: aOR, 9.00, 95% CI, 7.70-10.52), participants who used other tobacco products in the past 30 days (SHS: aOR, 1.36; 95% CI, 1.20-1.53; SHA: aOR, 1.33; 95% CI, 1.15-1.52), and those who live with someone who used e-cigarettes (SHS: aOR, 1.46; 95% CI, 1.24-1.72; SHA: aOR, 4.71; 95% CI, 3.91-5.68) were more likely to be exposed to SHS and SHA (Table). Youth who live with someone who used other tobacco products had higher odds of SHS exposure (aOR, 2.60; 95% CI, 2.34-2.90), and high school students had higher odds of SHA exposure (aOR, 1.49; 95% CI, 1.28-1.73) (Table).

**Table.** Prevalence of and Factors Associated With SHS and SHA Exposure in Public Places Among 20 189 US Middle and High School Students, National Youth Tobacco Survey, 2018

Characteristic^a^	Exposure			
	SHS		SHA	
	Weighted Prevalence, %	Adjusted OR (95% CI)^b^	Weighted Prevalence, %	Adjusted OR (95% CI)^b^
Sex				
Male	41.3	1 [Reference]	29.3	1 [Reference]
Female	56.0	1.96 (1.80-2.12)	37.1	1.68 (1.52-1.86)
School type				
Middle school	45.9	1 [Reference]	23.6	1 [Reference]
High school	50.8	0.98 (0.86-1.12)	40.9	1.49 (1.28-1.73)
Race/ethnicity				
Non-Hispanic white	53.7	1 [Reference]	39.4	1 [Reference]
Non-Hispanic black	40.6	0.64 (0.53-0.77)	19.2	0.47 (0.39-0.56)
Hispanic	44.5	0.75 (0.66-0.85)	29.1	0.70 (0.62-0.79)
Non-Hispanic other race^c^	44.5	0.81 (0.62-1.06)	29.2	0.76 (0.61-0.94)
Speak non-English language at home				
No	50.0	1 [Reference]	34.3	1 [Reference]
Yes	45.8	1.02 (0.91-1.13)	30.6	1.04 (0.93-1.17)
e-Cigarette use				
Never	43.4	1 [Reference]	22.5	1 [Reference]
Ever but not in the past 30 d	61.2	1.70 (1.52-1.92)	46.4	2.30 (2.02-2.62)
Past-30-d use	66.8	1.88 (1.64-2.17)	80.4	9.00 (7.70-10.52)
Past-30-d other tobacco use				
No	46.2	1 [Reference]	29.4	1 [Reference]
Yes	63.7	1.36 (1.20-1.53)	55.6	1.33 (1.15-1.52)
Lived with someone who used e-cigarettes				
No	46.5	1 [Reference]	28.9	1 [Reference]
Yes	69.6	1.46 (1.24-1.72)	75.5	4.71 (3.91-5.68)
Lived with someone who used other tobacco				
No	39.9	1 [Reference]	29.8	1 [Reference]
Yes	66.8	2.60 (2.34-2.90)	40.2	0.99 (0.88-1.12)

Abbreviations: e-Cigarette, electronic cigarette; OR, odds ratio; SHA, secondhand aerosol; SHS, secondhand smoke.

^a^ Missing cases include 1015 (5.0%) for SHS exposure, 989 (4.9%) for SHA exposure, 200 (1.0%) for sex, 143 (0.7%) for school type, 952 (4.7%) for speaking non-English language at home, 543 (2.7%) for e-cigarette use, and 398 (2.0%) for past-30-day use of other tobacco. Multiple imputation was used to address missing values. Ordinal logistic regression models using the original measures (ranging from 1-7) were fit as sensitivity analyses, and substantively similar results were found.

^b^ Weighted multiple logistic regressions estimating SHS and SHA exposures with participant characteristics.

^c^ Non-Hispanic other race includes Asian, American Indian or Alaska Native, and Native Hawaiian or other Pacific Islander.

## Discussion

Although 16 states and more than 800 municipalities have introduced laws to restrict e-cigarette use in 100% smoke-free or other venues, including schools, over the past few years,^3^ an increasing proportion of US youth reported exposure to SHA in public places in 2018 compared with previous years. This may be owing to the increase in youth using pod-based e-cigarettes and other devices,^2^ fewer vape-free policies than smoke-free policies,^3^ and fewer people who are willing to speak up against others vaping in public places.^4^ Beyond accelerating implementation of clean air laws, surveillance of SHA exposure trends, education about potential SHA harms for parents and youth, and interventions to reduce youth vaping are needed to protect young people from being exposed to all forms of tobacco product emissions, including from e-cigarettes.
